# Radiographic patterns on Chest X-ray as a supporting imaging tool in triaging of suspected Corona Virus Disease (COVID) patients

**DOI:** 10.12669/pjms.38.6.5279

**Published:** 2022

**Authors:** Tahira Nishtar, Nadeem Ullah, Fatima Sultan Ahmad, Sohana Rahim

**Affiliations:** 1 Dr. Tahira Nishtar, FCPS. Chairperson, Radiology Department, Lady Reading Hospital, Medical Teaching Institute, Peshawar, Pakistan; 2 Dr. Nadeem Ullah, FCPS Assistant Professor, Radiology Department, Lady Reading Hospital, Medical Teaching Institute, Peshawar, Pakistan; 3 Dr. Fatima Sultan Ahmad, FCPS. Registrar, Radiology Department, Lady Reading Hospital, Medical Teaching Institute, Peshawar, Pakistan; 4 Dr. Sohana Rahim, Resident Radiologist, Radiology Department, Lady Reading Hospital, Medical Teaching Institute, Peshawar, Pakistan

**Keywords:** Chest X-ray, COVID pneumonia, PCR, Modified Brixia Score

## Abstract

**Objective:**

To evaluate the radiographic patterns on Chest X-Ray (CXR) in accordance with Modified Brixia Scoring as supporting imaging tool in triaging of Corona Virus Disease (COVID-19) pneumonia.

**Methods:**

In this cross-sectional study, chest radiographs of suspected COVID patients at emergency triage, Lady Reading Hospital (LRH) from April 18^th^ to July 22^nd^ 2020 were evaluated for patterns of COVID pneumonia and scored in accordance with modified Brixia score. Each zone was categorized as score of “one” for interstitial pattern, “two” for mixed interstitial /alveolar pattern and “three” for alveolar pattern. Radiographic patterns consistent with COVID pneumonia or patients having strong clinical suspicion were advised Polymerase Chain Reaction (PCR) tests.

**Results:**

Total of 2,225 individuals were screened for patterns of COVID-19 pneumonia on chest radiograph. Out of these 1465(65.8%) had normal chest radiograph and 760(34.2%) had abnormal findings. Out of the total, 648 suspected COVID patients were selected for PCR. The radiographic patterns ranged from mixed interstitial/alveolar pattern in 261(40.3%) patients, alveolar pattern in 231(35.6%), interstitial pattern in 87(13.4%), pleural effusion in 12(1.9%), other findings in 5(0.8%) while 52(8%) suspected Covid patients had normal radiographs. The PCR was positive in 326(50.3%), negative in 100(15.4%) and inconclusive in 60(9.3%) while 162(25%) were lost to follow up. Amongst the 52 suspected Covid patients having normal chest radiographs, 10 were positive on PCR, 21 negative, seven suspected and two inconclusive, while 12 were lost to follow up.

**Conclusion:**

Chest radiograph is used for triaging of suspected COVID pneumonia patients in emergency settings. It assesses the severity of disease according to modified Brixia scoring for treatment plan.

## INTRODUCTION

Chest radiograph manifestations can supplement parts of limitations of PCR assay. The request for chest radiographs has grown exponentially and proportionally with the number of patients visiting the emergency department. A chest radiograph is performed in suspected or confirmed patients, although less sensitive than Computed Tomography (CT), chest radiography is typically the first-line imaging modality used for patients with suspected COVID-19.^1^ For ease of decontamination, use of portable radiography units is preferred.^2^

Chest radiographs may be normal in early disease. Findings are most extensive 10-12 days after symptom onset.^3^,^4^ Frequent findings are airspace opacities, consolidation or ground glass opacification. The distribution is bilateral, peripheral, lower zone predominance. Pleural effusion is rare (3%).

### Rationale of Study

The study highlights the radiographic patterns in accordance with novel Modified Brixia Scoring in order to categorize Covid-19 pneumonia patients for treatment plan.

## METHODS

In this cross sectional study, chest X-rays of 2,225 patients presenting to emergency department of LRH during the first wave from April 18^th^ to July 22^nd^ 2020 were reviewed. Suspected COVID patients include patients at high risk for having COVID pneumonia based on clinical symptomatology, exposure to diagnosed COVID-19 pneumonia patients or abnormal chest radiographic patterns consistent with covid pneumonia. Patients of all ages and both genders were included in this study. Patient selection was consecutive, in accordance with the emergency department guidelines. Staff safety regarding protection and adequate disinfection protocols were followed.

The radiographs of patients were viewed by two qualified Consultant Radiologists with more than ten years post fellowship experience. The typical Covid radiographic findings consistent with Covid Radiological Assessment Data System (CO-RADS) include multifocal ground glass opacifications or consolidations, peripheral and basal in distribution. The probable non Covid findings include pneumothorax, lobar pneumonia with or without pleural effusion and pulmonary edema.

The scoring system used at our institution to assess CXR as supporting tool in Covid pneumonia is zonal in accordance with modified Brixia scoring.^5^

Radiologically the lungs are divided into six zones on chest radiograph as following;

• Upper Zones (zone A and D) – above the inferior wall of the aortic arch,
• Mid Zones (zone B and E)– below the inferior wall of the aortic arch and above the inferior wall of the right inferior pulmonary vein (at the level of the hilum)
• Lower Zones (zone C and F)–below inferior wall of the right inferior pulmonary vein (lung bases).

For each zone involved a score of “one” is assigned for interstitial changes, “two” for mixed interstitial with mild alveolar pattern and “three” for prominent alveolar shadowing with ground glass opacification or consolidation, especially peripheral and patchy in distribution. Score of the predominant pattern was determined for each zone in a chest radiograph and sum of all the zones involved was calculated. Descriptive analysis of modified Brixia predominant radiographic patterns and compared with PCR result. Data was collected after approval from Institution Ethics Review Board on March 2020 (Reference Number 88 /LRH). Data was analyzed by SPSS-22. Frequency and percentages of gender and radiographic pattern were calculated. Post stratification chi square test was applied to radiographic patterns and PCR result, and to assess association of modified Brixia scores with PCR results Oneway Anova test was applied keeping p-value of 0.001 to be statistically significant.

## RESULTS

A total of 2,225 individuals were screened with chest radiograph as a supporting tool in triaging of COVID pneumonia patients. Out of these 1336 (60%) were male, while 889(40%) were female patients.

One thousand four hundred sixty-five (65.8%) radiographs were reported as normal. The remainder 760(34.2%) radiographs had abnormal radiographic features.

Amongst the individuals screened by chest radiographs, 648 were selected for PCR on clinical suspicion and radiographic findings. The major radiographic abnormalities ranging from mixed interstitial with alveolar pattern in 261(40.3%) patients, predominant alveolar pattern in 231(35.6%) patients, while 87(13.4%) presented with interstitial pattern only. Other patterns in patients included pleural effusion in 12(1.9%) and 5(0.8%) patients had findings such as lung nodules, pneumoconiosis, pneumothorax etc, as listed in Table-I, while 52(8%) suspected Covid patients had normal radiographs.

**Table-I:** Radiographic Predominant Patterns in suspected COVID patients.

Predominant Radiographic Patterns	Frequency	Percentages
Normal	52	8.0%
Interstitial Pattern	87	13.4%
Interstitial and Alveolar Pattern	261	40.3%
Alveolar Pattern	231	35.6%
Pleural Effusion	12	1.9%
Other findings	5	0.8%
Total	648	100%

**Fig 1:**
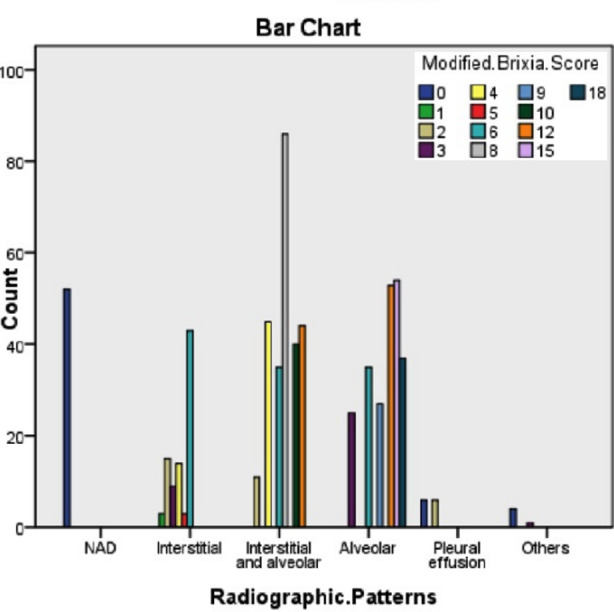
Bar Graph modified Brixia Scores in Predominant Radiographic Patterns.

The radiographs were scored from zero with no abnormality to maximum 18 in bilateral diffuse multilobar ground glass opacification/consolidation. Pleural effusions and other abnormalities were scored zero, even if clinically suspected for COVID infection in which case PCR analysis was advised. Application of modified Brixia score to individual chest radiographs’ is shown in Fig.1.

Of the 648 patients advised PCR test, 326(50.3%=326/648) had a positive result, 100(15.4%) were negative, 60(9.3%) patients had inconclusive results hence result categorized as suspected and 162(25%) were lost to follow-up due to factors such as improper sampling or issues with transportation of sample due to offsite testing facility.

The individual radiographic pattern and PCR result are compared and displayed in Table-II. The majority of PCR positive patients had mixed interstitial alveolar or predominant alveolar distribution of disease on chest radiographs. It is pertinent to mention here that amongst the 52 suspected covid patients having normal chest radiographs 10 showed positive result on PCR, 21 were negative, seven were suspected and two were inconclusive, while 12 were lost to follow up.

**Table-II:** Comparison of Radiographic pattern with PCR result.

Radiographic Pattern	PCR Result					

	Positive	Negative	Suspected	Inconclusive	Lost to follow-up	Total
NAD	10	21	7	2	12	52
Interstitial	44	16	4	1	22	87
Interstitial and Alveolar	147	30	20	3	61	261
Alveolar	118	28	17	3	65	231
Pleural Effusion	7	3	1	0	1	12
Others	0	2	2	0	1	5
Total	326 (50.3%)	100 (15.4%)	51 (7.9%)	9 (1.4%)	162 (25.0%)	648 (100%)

Chi square test was applied to radiographic patterns and PCR result. The p-value is less than 0.001 and statistically significant. The Anova test was applied to compare modified Brixia scores and PCR results that was found to have a p-value of 0.001 and statistically significant.

## DISCUSSION

Role of imaging in COVID-19 Pneumonia is multifactorial such as screening, diagnosis, severity assessment and monitoring the treatment response ie follow up.^6^ Provision of portable X ray^7^ as a supporting imaging tool for monitoring disease progression of admitted patients and dedicated radiographic unit to screen patients at the Emergency Triage, helped the clinicians with treatment plan .

Chest X ray scoring is used for assessing severity of disease, and it was first applied to Middle East Respiratory Syndrome (MERS), first identified in Saudi Arabia in September 2012. The imaging findings were very similar to Covid-19 pneumonia consisting of basal and peripheral ground glass opacification progressing to consolidation. The study of adult patients with MERS-CoV infection revealed that the chest radiographic score is an independent predictor of mortality.^8^

Various scoring systems have been introduced, including Radiographic Assessment of Lung Edema (RALE) method proposed by Warren et al,^9^ with maximum score of 4 for each lung, and ranging from 0 to a maximum score of 8, by summing up the individual scores of each lung. Another grading method used by Schalekamp et al,^10^ was introduced for COVID 19 pneumonia, after our data was collected. This scoring system divides lung into four zones and each zone is scored from 0 to 2 according to severity of radiographic COVID patterns, and the score to each zone are summed up. In our study we applied a modified method of the scoring suggested by Borghesi et al,^5^ (Brixia scoring). It is simple with total score of 18. Lungs are assessed by dividing into six zones on chest X-ray and the scoring system predicts disease severity relevant to clinical condition of patients. The original study for formulating the score included 100 patients hospitalized with confirmed Covid infection and the scoring reports ranged from 0 to 16 with a median of 6.5. The CXR score was significantly higher in patients who died than in those who were discharged from the hospital (*p* ≤ 0.002).^11^

In our study, scores were simplified on the bases of predominant pattern and the number of zones involved such as for interstitial lung disease pattern scored varied from 1 to 6, for mixed interstitial and alveolar pattern from 2 to 12, and for predominant alveolar pattern from 3 to 18. The scoring was in accordance with predominant chest radiographic pattern unlike the original Brixia scoring where each zone is categorized independently. For normal radiographs a score of zero was allocated. Our study stated 8.0% chest radiographs as normal. According to Cleverley et al^12^ 63% patients with Covid pneumonia had normal chest radiographs. In-keeping with a study by Kuo et al^13^ concluded in accordance with Fleischner Society recommendations, that screening chest radiography in young and middle-aged adults with asymptomatic or minimally symptomatic coronavirus disease is not indicated unless there is risk of deterioration. In our study amongst the abnormal radiographic patterns, predominant alveolar pattern was 35.6% and mixed interstitial alveolar opacification were 40.3%, collectively 75.9% with predominant lower lobe involvement. Khan et al^14^ emphasized abnormal radiographic patterns being 97% alveolar opacification with 98% lower lobe involvement. In their extensive study on 596 patients showed a comparative analysis of severe abnormal findings on X-rays with demographic and clinical characteristics showing a significantly higher proportion of X-ray severity in patients with shortness of breath (p-value <0.001) and chest pain (p-value 0.002).

Rousan et al.^15^ explained that peak severity score is reached on day 5 to 10. After 18 days, the lung abnormalities regress (50% GGO and 17% consolidation), with increase in the frequency of normal chest x-rays (33%) indicating a healing phase. A study by Majeed et al.^16^ included baseline 105 chest radiographs followed by serial follow up radiographs and concluded classic COVID radiographic findings more likely to have a positive PCR test, and indeterminate findings had negative PCR results. In our study similarly Chest X rays that showed classic COVID findings were majorly PCR positive (50.3%=326/648), however also including a few indeterminate radiographic findings like pleural effusion.

Durrani et al.^17^ conducted a study where radiographic findings of COVID positive PCR patients were described retrospectively without any scoring system. In a brief communication report by Bukhari et al^18^ stating the mechanism that cytokine storm determines the prognosis of the patients suffering from COVID19 leading to progressive abnormal radiological findings and prolonged hospital stay with bad prognosis.

Amongst the admitted patients with higher Modified Brixia Scores with predominant alveolar or mixed interstitial alveolar radiographic patterns who had moderate to severe symptoms, suffered longer hospital admissions, and equivocal outcome. Amongst the indigent patients 100 had radiographic findings consistent with Covid pneumonia but had PCR negative results, out of these 24 patients expired hence supporting mixed interstitial alveolar or predominantly alveolar opacification to have poor prognosis, even with negative PCR test. Whereas normal radiographs with negative PCR results had better prognosis therefore signifying role of chest radiograph as a supporting tool to assess severity of Pneumonia. A study by Kaleemi et al^19^ scored radiographs and correlated with outcome on a limited sample of 150 patients. Further quantitative assessment can be performed, Sayeed et al.^20^ concluded that CT chest severity scoring provides a good correlation with clinically evident disease burden.

### Limitations

At the time of first wave of Covid-19, our institution did not have onsite PCR testing facility with samples transported to offsite facilities. This factor led to inability to perform PCR in all individuals screened, however suspected COVID patients were advised PCR test and followed accordingly despite which 25% were lost to follow up.

## CONCLUSION

Chest radiograph is used for triaging of suspected Covid pneumonia patients in emergency settings. It assesses the severity of disease according to modified Brixia scoring for treatment plan.

### Authors’ Contributions:

**TN** Conception, manuscript writing & final approval.

**NU** Critical review and Interpretation of data. He is also the responsible for the accuracy of the study.

**FA** Results compilation, data analysis and discussion.

**SR** Data collection, results compilation and analysis..
